# No evidence of altered alveolar macrophage polarization, but reduced expression of TLR2, in bronchoalveolar lavage cells in sarcoidosis

**DOI:** 10.1186/1465-9921-11-121

**Published:** 2010-09-02

**Authors:** Maria Wikén, Farah Idali, Muntasir Abo Al Hayja, Johan Grunewald, Anders Eklund, Jan Wahlström

**Affiliations:** 1 Respiratory Medicine Unit, Department of Medicine, Karolinska Institutet, Stockholm, Sweden; 2 Department of Reproductive Immunology, Reproductive Biotechnology Research center, Avicenna Research Institute, Shahid Beheshti University, Evin, Tehran, Iran

## Abstract

**Background:**

Sarcoidosis is a granulomatous inflammatory disease, possibly of infectious aetiology. We aimed to investigate whether the degree of functional polarization of alveolar macrophages (AMs), or Toll-like receptor (TLR) expression, is associated with sarcoidosis or with distinct clinical manifestations of this disease.

**Methods:**

Total BAL cells (cultured four or 24 h in medium, or stimulated 24 h with LPS) from 14 patients and six healthy subjects, sorted AMs from 22 patients (Löfgren's syndrome n = 11) and 11 healthy subjects, and sorted CD4+ T cells from 26 patients (Löfgren's syndrome n = 13) and seven healthy subjects, were included. Using real-time PCR, the relative gene expression of IL-10, IL-12p35, IL-12p40, IL-23p19, CCR2, CCR7, iNOS, CXCL10, CXCL11, CXCL16, CCL18, CCL20, CD80, and CD86, and innate immune receptors TLR2, TLR4, and TLR9, was quantified in sorted AMs, and for selected genes in total BAL cells, while IL-17A was quantified in T cells.

**Results:**

We did not find evidence of a difference with regard to alveolar macrophage M1/M2 polarization between sarcoidosis patients and healthy controls. TLR2 gene expression was significantly lower in sorted AMs from patients, particular in Löfgren's patients. CCL18 gene expression in AMs was significantly higher in patients compared to controls. Additionally, the IL-17A expression was lower in Löfgren's patients' CD4+ T cells.

**Conclusions:**

Overall, there was no evidence for alveolar macrophage polarization in sarcoidosis. However, there was a reduced TLR2 mRNA expression in patients with Löfgren's syndrome, which may be of relevance for macrophage interactions with a postulated sarcoidosis pathogen, and for the characteristics of the ensuing T cell response.

## Introduction

Sarcoidosis is a systemic T helper 1 (Th1) inflammatory disease [1,2], primarily affecting the lungs. The hallmark of disease is non-caseating granulomas where macrophages are essential components. These cells are very heterogeneous, characterized by plasticity and functional polarization, with, as here named, M1 and M2 types, at the extremes of a continuum. Due to micro-environmental signals, such as cytokines, chemokines and Toll-like receptor (TLR) ligands, macrophages differ in receptor expression, cytokine and chemokine production, as well as effector function [3,4]. Classical activation, that is IFNγ, TNF and microbial products (e.g. lipopolysaccharide (LPS)), elicit the M1 form. This phenotype is characterized by high capacity to present antigens and high capacity to produce IL-12 (promoting Th1 responses) and IL-23 (promoting maturation and survival of IL-17 producing T cells), as well as microbicidal nitric oxide and reactive oxygen intermediates. In contrast, exposure to IL-4 or IL-13, immune complexes, and IL-10 induce the alternative activation leading to an M2 form and a relatively more Th2 response. High amounts of IL-10, but little IL-12 and IL-23, and abundant expression of non-opsonic receptors characterize this phenotype.

In addition to alveolar macrophages (AMs), sarcoidosis patients display increased numbers of CD4+ T lymphocytes in their lungs. Previously, a study from our group and others showed that these cells are highly positive for the chemokine receptor CXCR3 [5]. Further more, it has been shown that CXCR3 ligands, that is the M1 markers CXCL9, CXCL10 and CXCL11, seem essential in the pathogenesis of pulmonary sarcoidosis [6,7]. CXCL9 and CXCL10 appear to be involved in the active phase of the granulomatous response, whereas CXCL11, as well as CXCL10 and CXCL16 [8] and CCL20 [9], may play a role in the accumulation of Th1 cells, in the sarcoid lungs. However, the presence of a recently discovered T cell subset, the IL-17 producing Th17 cells, has to our knowledge not been investigated in sarcoidosis. Th17 cells have been implicated in autoimmune diseases [10] and is also important for combating extracellular pathogens [11].

The aetiology of sarcoidosis is still unknown. However, epidemiological studies and findings of DNA from mycobacteria [12] and propionibacteria [13] and mycobacterial antigens [14], in sarcoidosis tissue and lymph nodes indicate an infectious cause. This is further supported by the demonstration by us and others that mycobacterial antigens can elicit adaptive immune responses [15,16], which suggests a role for pattern-recognition receptors, such as TLRs, in the pathogenesis. TLRs are expressed on antigen presenting cells, and as key mediators of innate host defence these receptors are involved in recognizing several molecules derived from microbes of different kinds. For example, mycobacteria contain ligands for TLR2 and TLR4.

There is a considerable variation in the clinical manifestations of sarcoidosis. Patients who present with Löfgren's syndrome, i.e. erythema nodosum and/or ankle arthritis, fever and bilateral hilar lymphadenopathy with or without parenchymal infiltration, are characterized by an acute onset and a good prognosis and usually recover spontaneously within two years. They are often HLA-DRB1*03 positive. Other patients, here named non-Löfgren's syndrome patients, often have HLA-DRB1*14 or 15 haplotype, show an insidious disease onset with dry cough and fatigue, and are at risk of developing pulmonary fibrosis [17]. The aim of this study was to elucidate if the degree of BAL cell polarization, with regard to M1 and M2 associated cytokines, chemokines and chemokine receptors, may be associated with sarcoidosis, or related to clinical manifestations of sarcoidosis. In addition, we studied the expression of the innate immune receptors TLR2 and TLR4.

## Methods

### Study subjects

Sarcoidosis patients included in this study were consecutive patients referred to the Respiratory Medicine Unit (Karolinska University Hospital, Stockholm, Sweden) for investigation. All patients were diagnosed with pulmonary sarcoidosis as determined by symptoms, chest radiography and pulmonary function tests and the diagnosis was established using the criteria by the World Association of Sarcoidosis and other Granulomatous Disorders (WASOG) [18]. Written informed consent was obtained from all subjects, and the Regional Ethical Review Board approved the study.

A total of 36 sarcoidosis patients and 17 healthy subjects participated in this study. Total bronchoalveolar lavage (BAL) cells of 14 sarcoidosis patients (median age 40 yrs, p25-p75 = 34-67 yrs; nine males and five females), of which one had Löfgren's syndrome, and six healthy subjects (median age 28 yrs, p25-p75 = 26-39 yrs; two males and four females), were cultured in medium (four or 24 h), or stimulated with LPS (24 h). In addition, AMs were sorted (see *BAL and preparation of cells*), from a total of 22 sarcoidosis patients separate from the above (median age 38 yrs, p25-p75 = 32-47 yrs; nine males and 13 females), of which 11 had Löfgren's syndrome, and 11 healthy subjects (median age 27 yrs, p25-p75 = 26-30 yrs; six males and five females). Only one patient was undergoing treatment with a non-steroid anti-inflammatory drug (diclofenac); the rest were untreated. All patients were fasting before BAL was performed, and none had signs of infections. All healthy subjects had a normal chest X-ray. Clinical and BAL fluid characteristics, of sarcoidosis patients and healthy subjects, are given in Table 1 and Table 2, respectively. Significant differences in clinical parameters between patients and healthy subjects are indicated in Table 1. There where no significant differences in lung function parameters or BAL cell differential counts between patients with or without Löfgren's syndrome. In addition, we used stored cDNA from CD4+ T cells, from a total of 26 non-smoking sarcoidosis patients, of which 13 had Löfgren's syndrome, and seven non-smoking healthy subjects. These CD4+ T cells where previously FACS-sorted to be included in two other studies from our group, where a detailed patient characterization can be found [19,20].

**Table 1:** Characterization of patients

	SORTED ALVEOLAR MACROPHAGES			TOTAL BAL CELLS
	**All sarcoidosis** **patients (n = 22)**	**Löfgren's** **syndrome (n = 11)**	**Non-Löfgren's** **syndrome (n = 11)**	**All sarcoidosis** **patients (n = 14)**

Sex, male/female	9/13	3/8	6/5	9/5
Age, yr	38 (32-47)**	38 (32-39)^#^	40 (31-55)^##^	40 (34-67)^φ^
Smoker (yes/ex/never)	1/7/14	0/3/8	1/4/6	1/4/8 (1 nd)
X-ray stage (0/I/II/III/IV)	0/11/7/3/1	0/9/2/0/0	0/2/5/3/1	2/3/7/1/0 (2 nd)
Oral steroid treatment	0	0	0	0
				
**BAL analyses**				
% Recovery	73 (61-77)	73 (63-76)	72 (59-79)	68 (59-71)
% Viability	94 (92-96)	94 (92-98)	94 (92-95)	95 (92-97)
Cell concentration (*10^6^/L)	197 (130-250)**	173 (131-225)^#^	213 (119-296)^##^	203 (153-257)
Total cell number (*10^6^)	30 (24-38)**	31 (25-38)^#^	27 (23-45)^#^	32 (24-36)
				
**BAL differential cell counts**				
% Macrophages	74 (62-79)*	74 (62-79)	73 (61-81)	69 (52-76)
% Lymphocytes	24 (18-38)**	23 (19-37)^#^	26 (15-39)	31 (22-47)^φ^
% Neutrophils	0.9 (0-6-2.5)	1.0 (0.8-3.6)	0.8 (0.5-2.0)	1.0 (0.6-2.0)
% Eosinophils	0.3 (0-1.1)	0.2 (0-1.0)	0.5 (0-1.6)	0.4 (0-1.3)
				
CD4/CD8 ratio	7.9 (3.6-16)	8.5 (4.7-12)	6.7 (2.0-19)	8.5 (2.4-15)
				
**Pulmonary function tests**				
VC	89 (81-97)	89 (81-96)	87 (75-98)	87 (79-100)
FEV_1_	88 (84-99)	87 (85-100)	94 (82-99)	85 (79-96)
DL_CO_	86 (73-94)	86 (73-95)	84 (73-93)	85 (75-99)

Data are shown as median (p25-p75)

nd = not determined

* *p *< 0.05, ** *p *< 0.01, versus sorted macrophages healthy subjects

^# ^*p *< 0.05, ^##^*p *< 0.01, versus sorted macrophages healthy subjects

^φ ^*p *< 0.05, versus total BAL cells healthy subjects

Pulmonary function tests:

VC: Vital capacity, % of reference value

FEV_1_: Forced expiratory volume in one second

DL_CO_: Diffusing capacity of the lung for carbon monoxide

Values shows % of predicted

**Table 2:** Characterization of healthy subjects

	SORTED ALVEOLAR MACROPHAGES	TOTAL BAL CELLS
	**Healthy subjects** **(n = 11)**	**Healthy subjects** **(n = 6)**

Sex, male/female	6/5	2/4
Age, yr	27 (26-30)	28 (26-39)
Smoker (yes/ex/never)	0/1/10	1/0/5
		
**BAL analyses**		
% Recovery	74 (64-78)	70 (62-82)
% Viability	96 (95-98)	95 (93-98)
Cell concentration (*10^6^/L)	91 (69-131)	127 (86-187)
Total cell number (*10^6^)	18 (12-22)	20 (12-32)
		
**BAL differential cell counts**		
% Macrophages	84 (79-89)	87 (64-93)
% Lymphocytes	14 (9.0-20)	11 (5.1-33)
% Neutrophils	2.0 (0.7-2.8)	1.9 (1.0-2.1)
% Eosinophils	0.2 (0-1.2)	0.3 (0-0.9)

Data are shown as median (p25-p75)

### BAL and preparation of cells

BAL was performed as previously described [21]. Briefly, under local anesthesia, a flexible fiber-optic bronchoscope (OBF Type 1TR; Olympus Optical Co., Tokyo, Japan) was passed transorally and wedged into the middle-lobe bronchus. Sterile phosphate-buffered saline (PBS) solution was at 37°C instilled in five aliquots of 50 ml each. After instillation the fluid was aspirated and collected in a siliconised plastic bottle kept on ice. The BAL fluid was strained through a Dacron net (Millipore, Cork, Ireland) and centrifuged at 400 × g for 10 min at 4°C, and the pellet was resuspended in RPMI-1640 medium (Sigma-Aldrich, Irvin, UK). Cells were counted in a Bürker chamber and the viability was determined by trypan blue exclusion. Ratio of CD4/CD8 T cell were determined by flow cytometric analysis (FACSCalibur and FACSCanto, Becton Dickinson, Mountain View, CA, USA) using monoclonal anti-CD3, anti-CD4, and anti-CD8 antibodies (Dako Cytomation Norden AB, Solna, Sweden), as previous described [22]. Using FACSVantage (BD Biosciences, Mountain View, CA, USA), AMs (recovery 0.02-2.8 × 10^6^) were sorted from total BAL cells, based on cell size (forward scatter) and granularity (side scatter). CD4+ T cells where sorted as previously described [20].

### In vitro stimulation of total BAL cells

Total BAL cells were pelleted and resuspended in complete medium (10^6 ^cells/1 ml); RPMI-1640 medium (Sigma-Aldrich, Irvine, U.K.), supplemented with 1% penicillin streptomycin (Invitrogen Corporation, Paisley, Scotland), 1% L-glutamine (Sigma-Aldrich, Irvine, U.K.) and 2% heat-inactivated human AB serum (Sigma-Aldrich, Schnelldorf, Germany), for four or 24 h, or stimulated with the classical macrophage activator and TLR4 ligand LPS (1.6 μg/ml, *Salmonella enterica serotype abortus equi*, Sigma-Aldrich, Schnelldorf, Germany) for 24 h [23], in 96-well U-bottom tissue culture plates at 37°C in humidified atmosphere of 5% CO_2 _in air.

### RNA extraction and cDNA synthesis

Total RNA was extracted *via *the guanidium thiocyanate phenol-chloroform technique [24], using RNA Bee (Nordic Biosite, Stockholm, Sweden). Briefly, 1-2 ×10^6 ^cultured total BAL cells or AMs were incubated with 300-600 μL RNA Bee for 10 min at room temperature, and then stored at -70°C until use. After thawing, 60-120 μL chloroform was added to each sample, which was shaken vigorously and kept on ice for 5-10 min. After centrifugation at 12.000 × g for 15 min at 4°C, the upper phase, containing RNA, was transferred to a new tube and at least an equal volume of ice-cold isopropanol was added. After an overnight incubation at -20°C, the samples were centrifuged at 12.000 × g for 20 min at 4°C. The RNA pellet was washed in 75% ice-cold ethanol (at 7500 × g for 10 min at 4°C), followed by air drying for 10-15 min. The pellet was thereafter dissolved in 20 μL autoclaved ultra clean water.

To synthesize cDNA, 1 μg of total RNA was incubated in the presence of 20 mM random hexamers primers (Pharmacia Biotech, Uppsala, Sweden) and 200 units Superscript™II RNase H^- ^Reverse transcriptase (Invitrogen, Lidingo, Sweden) for 10 min at room temperature and then 45 min at 40°C, followed by 5 min at 95°C to inactivate the enzyme. The cDNA samples were stored at -20°C until use.

### Analysis of gene expression by real-time PCR

By real-time PCR, the relative gene expression of M1 associated markers (IL-12p35, IL-12p40, IL-23p19, CCR7, iNOS, CXCL10, CXCL11, CXCL16, CCL20, CD80, CD86), M2 associated markers (IL-10, CCR2, CCL18), and innate immune receptors (TLR2, TLR4, TLR9), was quantified, using ABI Prism 7700 Sequence Detection System (Applied Biosystems, Foster City, CA, USA), as was the expression of IL-17A.

For human IL-10 and IL-12p40 (both according to [2]), a PCR reaction was set up in a 25-μl reaction volume as previously described [2]. Briefly, 1 × Taqman buffer II, 0.5 U Ampli-Taq gold, MgCl_2 _concentration optimized for each cytokine (Applied Biosystems), 0.5 mM deoxyribonucleoside triphosphate (Amersham Bioscience, Uppsala, Sweden), 5.0 pmol of each forward and reverse primer and 2.5 pmol probe (all from Cybergene AB, Stockholm, Sweden). The human assay-on-demand products for IL-12p35 (Hs00168405_m1), IL-23p19 (Hs00372324_m1), CCR7 (Hs00171054_m1), iNOS (Hs00167257_m1), CXCL10 (Hs00171042_m1), CXCL11 (Hs00171138_m1), CXCL16 (Hs00222859_m1), CCL20 (Hs00355476_m1), CD80 (Hs00175478_m1), CD86 (Hs00199349_m1), CCR2 (Hs00356601_m1), CCL18 (Hs00268113_m1), TLR2 (Hs00152932_m1), TLR4 (Hs00152939_m1), TLR9 (Hs00152973_m1), IL-17A (Hs00174383_m1), and universal master mix were purchased commercially (Applied Biosystems, Foster City, CA, USA).

2 μl of the cDNA (diluted in autoclaved ultra clean water according to pilot experiments) where put in each well on an optical 96-well reaction plate (Applied Biosystems, Foster City, CA, USA) together with gene mix. Human β-actin (hs9999903_m1, or according to [2]) was used as a housekeeping gene to normalize the values of other genes.

The PCR condition was a followed: an initial period of 2 min at 50°C and 10 min at 95°C, followed by 40 cycles involving denaturation at 95°C for 15 sec and annealing/extension at 60°C for 1 min. All samples were run in duplicates and the mean values were calculated.

For relative quantification of expression of cytokine genes in cultured total BAL cells and in sorted macrophages, the following arithmetic formula was used: 2^-ΔΔCT ^[25]. The amount of target gene was normalized to the housekeeping gene (β-actin) and the relative expression of target genes in cultured total BAL cells was calculated in relation to the mean values of target gene expression in healthy subjects after 24 h of incubation in medium alone. The relative expression of target genes in sorted macrophages was calculated in relation to the mean values of target gene expression in the healthy subject group. PCR amplification efficiencies for both the endogenous control (β-actin) and target genes were tested through serially diluting a cDNA sample and showing that the CT difference between the target and endogenous control remained constant.

### Statistical methods and data management

The Mann-Whitney U-test was used for comparison of relative gene expression between sarcoidosis patients and healthy subjects. One-way ANOVA (Kruskal-Wallis), was used for comparison of relative gene expression between patient subgroups (with and without Löfgren's syndrome), and healthy subjects. In the case of a statistically significant result in the ANOVA, statistical comparisons were made by use of the post-hoc test proposed by Dunn to control for multiplicity. The within group analysis (cell culture 24 h with or without LPS) was made by use of the Wilcoxon Signed Rank Test. The Spearman rank correlation coefficient was used in order to test independence between variables. A *p *value of <0.05 was considered as significant. The study employs multiple hypotheses testing, where each hypothesis was analyzed separately and the existence of patterns in and the consistency of the results were considered in the analysis. All analyses were carried out by use of the computer program GraphPad PRISM 4.03 (GraphPad Software Inc., San Diego, CA, USA).

## Results

The relative gene expression of several M1 and M2 associated markers was quantified in cultured total BAL cells and sorted AMs from sarcoidosis patients, with or without Löfgren's syndrome, and healthy subjects. In addition, the expression of selected TLRs was measured in sorted AMs, and IL-17A was measured in sorted CD4+ T cells.

### Gene expression after culturing and stimulation of total BAL cells

**Figure 1:**
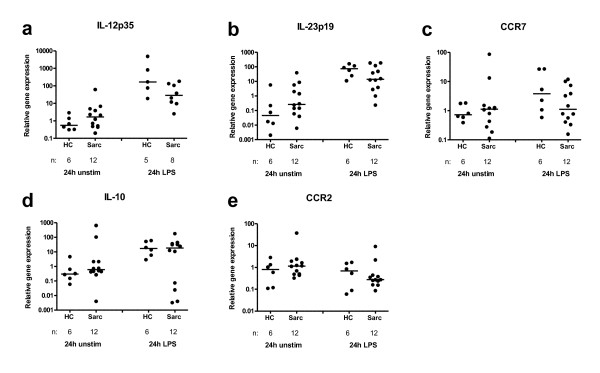
**Total BAL cell expression of the M1 markers IL-12p35 (a), IL-23p19 (b) and CCR7 (c), and the M2 markers IL-10 (d) and CCR2 (e) mRNA, in healthy controls (HC) and sarcoidosis patients (Sarc), cultured in medium (24 h), or stimulated with LPS (24 h)**. Horizontal lines depict median values. (n: number of individuals analyzed.)

First we studied the relative gene expression of the typical M1 markers, IL-12p35 (Fig. 1a), IL-23p19 (Fig. 1b), CCR7 (Fig. 1c), and M2 markers, IL-10 (Fig. 1d) and CCR2 (Fig. 1e), in total BAL cells of sarcoidosis patients and healthy subjects after culturing in medium for four or 24 h, or after 24 h of LPS stimulation. The number of markers and stimulating conditions that were investigated was limited by the amount of cells available for the study.

**Figure 2:**
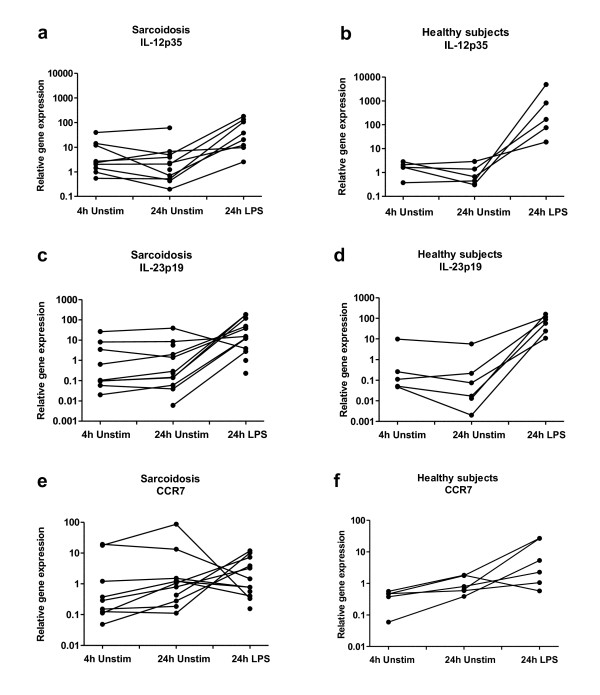
**Intra-individual comparisons of relative gene expression of the M1 markers IL-12p35 (a, b), IL-23p19 (c, d) and CCR7 (e, f) in total BAL cells cultured in medium (four or 24 h), or stimulated with LPS (24 h), in sarcoidosis patients (left columns) and healthy controls (right columns)**.

**Figure 3:**
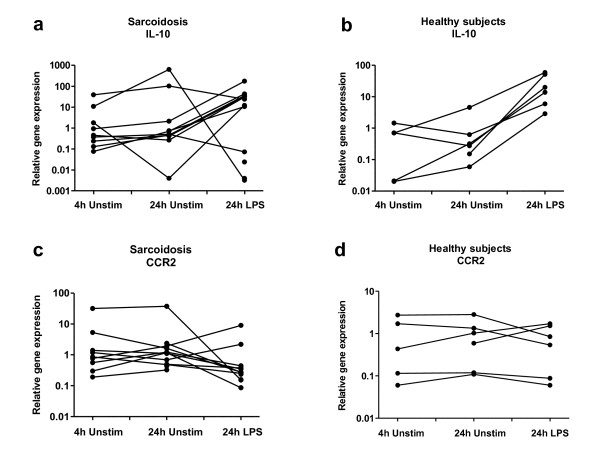
**Intra-individual comparisons of relative gene expression of the M2 markers IL-10 (a, b) and CCR2 (c, d) in total BAL cells cultured in medium (four or 24 h), or stimulated with LPS (24 h), in sarcoidosis patients (left columns) and healthy controls (right columns)**.

In total BAL cells we found no evidence of different M1/M2 polarization between patients and controls, either after 24 h incubation in medium alone or with LPS stimulation (Fig. 1). There were only minor differences in gene expression after 4 h culturing in medium alone (data not shown) compared to after 24 h culturing in medium. Due to limitations in the number of cells available, gene expression after all three culture conditions could not be studied in all individuals. The exact numbers of included patients or healthy subjects are indicated under each plot (Fig. 1). Data from the patients and healthy subjects, where it was possible to study all three culture conditions, is shown in Figure 2 and 3. LPS upregulated IL-12p35 and IL-23p19 in both groups (this was not statistically significant for IL-12p35 in controls, probably because of too few individuals). When comparing the magnitude of upregulation (the differences between relative gene expression after 24 h incubation in medium alone or with LPS stimulation), we noted tendencies of lower upregulation in sarcoidosis patients. In addition, LPS significantly upregulated IL-10 in healthy controls.

It was noted that patients with the highest gene expression after 24 h of culturing of total BAL cells in medium alone had a large decrease in production after LPS stimulation. The two patients showing that pattern had extrathoracic disease, pronounced BAL lymphocytosis, and high BAL CD4/CD8 ratio.

Since there were only total BAL cells from one patient with Löfgren's syndrome, no subgroup comparison was done.

### Screening of M1 and M2 associated markers in alveolar macrophages

We next freshly isolated AMs, using flow cytometric cell sorting, and measured the relative gene expression of a wide range of different M1 and M2 markers.

**Figure 4:**
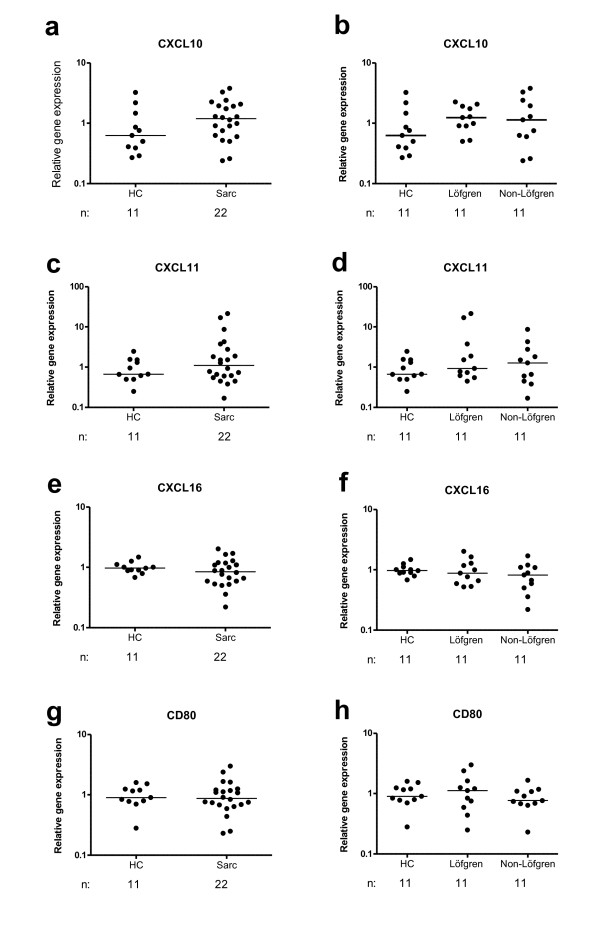
**Alveolar macrophage expression of the M1 markers CXCL10 (a, b), CXCL11, (c, d), CXCL16 (e, f), and CD80 (g, h) mRNA of (a, c, e, g) healthy controls (HC) and sarcoidosis patients (Sarc), and (b, d, f, h) HC, Löfgren's syndrome patients and Non-Löfgren's syndrome patients**. Horizontal lines depict median values. (n: number of individuals analyzed.)

We did not find any significant differences between patients and healthy subjects, and no difference between patients subgroups with regard to the M1 markers CXCL10, CXCL11, CXCL16, CD80 (Fig. 4), CD86 and CCL20 (data not shown). The expression of IL-12p40, IL-23p19, CCR7 and iNOS from most of the samples were below the detection limit (data not shown).

**Figure 5:**
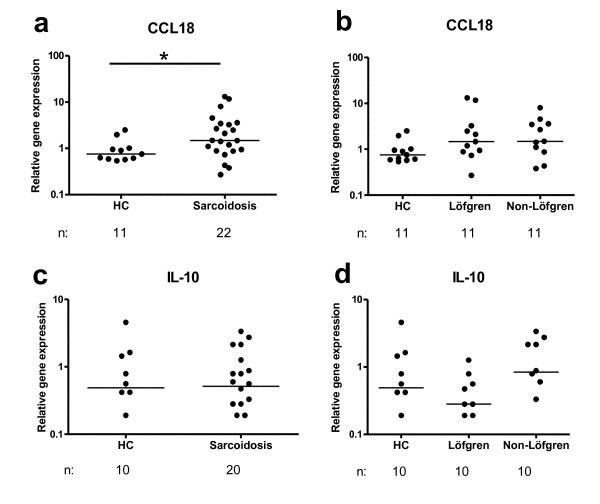
**Alveolar macrophage expression of the M2 markers CCL18 (a, b) and IL-10 (c, d) mRNA in (a, c) healthy controls (HC) and sarcoidosis patients, and in (b, d) HC, Löfgren's syndrome patients and Non-Löfgren's syndrome patients**. Horizontal lines depict median values. * *p *< 0.05. (n: number of individuals analyzed.)

As shown in Figure 5a, the relative gene expression of CCL18 (an M2 associated marker) was significantly higher in sarcoidosis patients compared to healthy subjects (*p *= 0.034). Yet, there was no difference between patient subgroups (Fig. 5b). The expression of CCL18 was positively correlated with the percentage of lymphocytes in BAL fluid (data not shown). There were no differences between patients and healthy subjects regarding IL-10 (Fig. 5c) or CCR2 (data not shown).

### TLR mRNA expression in alveolar macrophages

**Figure 6:**
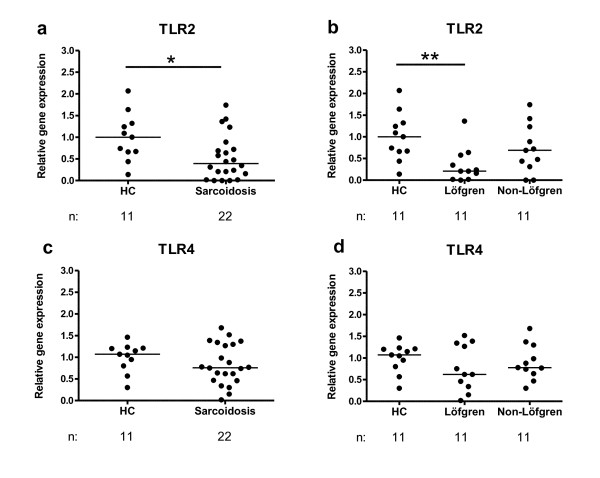
**Expression of TLR2 (a, b) and TLR4 (c, d) mRNA in alveolar macrophages in (a, c) healthy controls (HC) and sarcoidosis patients, and in (b, d) HC, Löfgren's syndrome patients and Non-Löfgren's syndrome patients**. Horizontal lines depict median values. * *p *< 0.05, ** *p *< 0.01. (n: number of individuals analyzed.)

There was a significantly reduced TLR2 gene expression in sorted AMs from sarcoidosis patients compared to healthy subjects (*p *= 0.024) (Fig. 6a). Furthermore, when studying patient subgroups, a significantly lower TLR2 expression was observed in patients with Löfgren's syndrome compared to healthy subjects (*p *= 0.0058) (Fig. 6b). The relative gene expression of TLR4 (Fig. 6c, d), and TLR9 (data not shown), did not differ significantly between the groups.

### IL-17A mRNA expression in sorted CD4+ T cells

**Figure 7:**
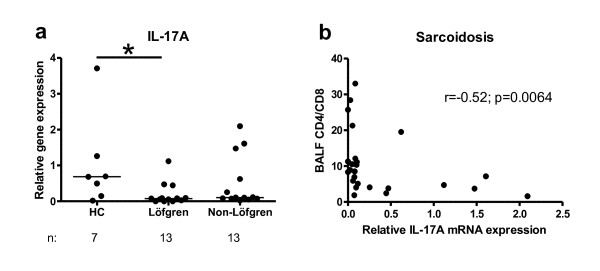
**Expression of IL-17A mRNA in BAL fluid CD4+ T lymphocytes in healthy subjects (HC), Löfgren's syndrome patients and Non-Löfgren's syndrome patients (a)**. Horizontal lines depict median values. * *p *< 0.05. **Correlation between IL-17A mRNA expression and BALF CD4/CD8 ratio (b).**

The tendency to a less pronounced upregulation of IL-23p19 expression in sarcoidosis patients after LPS stimulation, made us believe that this could have an impact on the induction of Th17 cells. We therefore analysed IL-17A expression in FACS-sorted CD4+ T cells. As shown in Figure 7a, the relative gene expression of IL-17A was lower in patients, statistically significant in patients with Löfgren's syndrome. However, this needs to be interpreted with caution since the significance is lost if the outlier with the highest IL-17 mRNA expression among the healthy controls is omitted. Furthermore, in patients there was a negative correlation of IL-17A expression with CD4/CD8 BALF ratio (Fig. 7b).

Since there is a reciprocal interconnection between the development of Th17 cells and Treg cells [26], and Th17 cells can be controlled by Foxp3+ T regulatory cells [27], we attempted to investigate the balance between these two subsets by correlating IL-17 mRNA expression with that of Foxp3, using data for Foxp3 mRNA expression in the same samples of sorted CD4+ BAL T cells already included in a previous study [19]. However, no correlation was found, either in total patients or in subgroups (Löfgren and non-Löfgren) (data not shown).

## Discussion

In the present study we aimed to investigate whether functional polarization of alveolar macrophages was associated with sarcoidosis, or with patient subgroups. This was found not to be the case, although there was a higher expression of the fibrosis-associated marker CCL18 in AMs in the whole group of patients. In addition, we studied the expression of pattern-recognition receptors TLR2 and TLR4, and found that AMs from patients, in particular those with Löfgren's syndrome, had a lower expression of TLR2.

In contrast to our negative findings regarding macrophage polarization, previous studies have demonstrated higher expression of the M1 markers CXCL10 [6], CXCL11 [7] and CXCL16 [8] in sarcoidosis patients compared to healthy subjects. However, in those studies AMs or total BALF cells were cultured in medium over night before gene expression were measured. In the present study we focused on freshly isolated AMs. These differences in results may arise because a certain degree of stimulation is needed to make macrophages express cytokine genes and reveal their functional potential. E.g. it has been shown that adherence to plastic can by itself cause macrophage activation and cytokine production [23]. A lack of such *in vitro *activation in our study may also explain why the mRNA levels of some genes such as IL-2p35 were often below the detection limit, in agreement with previous studies [28].

Similarly to a previous study [29], we here demonstrate an increased gene expression of CCL18 in AMs of sarcoidosis patients as compared to healthy subjects. However, another study found no differences in CCL18 mRNA expression between patients and controls, yet in that study total BAL fluid cells and not sorted macrophages were studied [30]. Functional studies of CCL18 has indicated a profibrotic role for this chemokine as part of a positive feedback loop between AMs and fibroblasts [29]. CCL18 has been reported to be an indicator of pulmonary fibrosis since BAL cells of sarcoidosis patients with X-ray stage IV produce higher levels of CCL18 compared to a lower X-ray stage [29]. There have also been findings of high levels of CCL18 in patients with other fibrosing lung disorders [31,32]. In addition, plasma levels of CCL18 have been suggested to be a marker of disease activity [33]. We did not find any correlations with X-ray stage, or lung function parameters in our study. However, the majority of our patients had X-ray stage I or II, with only one at X-ray stage IV. Neither did we observe any difference in CCL18 expression between patients with Löfgren's syndrome or not. We found, however, that the expression of CCL18 was positively correlated with the percentage of lymphocytes. Therefore, CCL18 may act mainly as a T cell attractant, preferentially of naïve T cells [34], in the early stage of disease, while the profibrotic role of CCL18 may only be important in more advanced disease.

We found that AMs of patients are characterized by a lower gene expression of TLR2 compared to healthy subjects. This is in contrast to our previous report of higher expression of TLR2 and TLR4 receptors on blood monocytes [35], although it should be noted that different cell types were studied (monocytes *vs*. macrophages) and different techniques used (cell surface receptor expression *vs*. mRNA expression). One explanation for this difference in TLR expression between lung and blood could be differences in exposure to various stimuli known to affect TLR expression. For example, TLR ligands are able to up- or downregulate TLR mRNA expression depending on dose and time [35-37] In addition, the cell surface level of TLR2 was found to be increased by some cytokines, and decreased by others [38]. E.g. IFNγ and TNF downregulated TLR2 levels on human monocytes, although macrophages were not studied. Therefore, the totality of different inflammatory mediators present in different compartments are likely to determine local TLR expression. Moreover, the TLR2 down-regulation was mainly seen in patients with Löfgren's syndrome, possibly indicating that these patients respond to a particular ligand that specifically binds to TLR2.

The finding of a tendency to a smaller LPS-induced increase in IL-23 expression in sarcoidosis patients raised the question whether Th17 cells may be differently affected in sarcoidosis patients compared to healthy subjects. We found that the relative gene expression of IL-17 in CD4+ T cells was reduced in sarcoidosis patients, statistically significant (although weakly so) in patients with Löfgren's syndrome compared to healthy subjects. The reason for this is not clear, but it may be related to findings in recent studies reviewed in [39] showing that the Th17 phenotype is unstable, and via T-bet induction can be converted to a Th1 phenotype. It may be speculated that the Th1-inducing lung milieu characteristic of sarcoidosis has such an effect on Th17 cells. Furthermore, we found a negative correlation between BALF CD4+ T cell IL-17A expression and BALF CD4/CD8 ratio in sarcoidosis, suggesting that the alveolitis seen in patients is associated with an influx of CD4 T cells with on average merely little production of IL-17. The roles of IL-23 and IL-17 in sarcoidosis clearly merit further investigation.

In a recent publication it was shown that smoking can affect macrophage polarization in the M2 direction [40]. Although there were very few current smokers among the patients and controls in our study, the smoking history differed when ex-smokers were taken into account. Previous smoking could potentially affect macrophage polarization, although that was not addressed in the above mentioned study. However, considering the difficulties in recruiting healthy controls for bronchoalveolar lavage obtaining a desired match with patients for factors such as smoking history and age is not practically feasible. Also, the above mentioned study was published after the samples for the present study were collected. We did not observe any obvious differences with regard to the expression of the different genes between individuals with different smoking status. Also, another report suggests that current, but not previous smoking, is important for macrophage gene expression. Using microarray analysis of human alveolar macrophages, it was found that despite the inclusion of ex-smokers in the group of non-smokers, there was complete segregation between non-smokers and smokers after cluster analysis of the 200 genes with the highest variance across smokers and non-smokers [41].

In conclusion, we did not find evidence for a difference in alveolar macrophage polarization in patients compared to healthy controls. The role of IL-23/IL-17 axis in sarcoidosis merits further study. The reduced TLR2 expression of AMs from patients with Löfgren's syndrome may suggest a distinct innate immune response to pathogens in these patients.

## Competing interests

The authors declare that they have no competing interests.

## Authors' contributions

MW participated in study planning, performed patient material collection, performed experiments and data analysis, and wrote the manuscript. FI performed patient material collection, performed experiments, and critically reviewed the manuscript. MH performed experiments. JG participated in study planning, recruitment of patients, and critically reviewed the manuscript. AE participated in study planning, recruitment of patients, and critically reviewed the manuscript. JW conceived the study and its design, performed data analysis, and supervised the writing of the manuscript. All authors read and approved the final manuscript.
